# Heterogeneous distribution of plankton within the mixed layer and its implications for bloom formation in tropical seas

**DOI:** 10.1038/srep11240

**Published:** 2015-06-11

**Authors:** Albert Calbet, Mette Dalgaard Agersted, Stein Kaartvedt, Malene Møhl, Eva Friis Møller, Søren Enghoff-Poulsen, Maria Lund Paulsen, Ingrid Solberg, Kam W. Tang, Kajsa Tönnesson, Dionysios E. Raitsos, Torkel Gissel Nielsen

**Affiliations:** 1 Institut de Ciències del Mar, CSIC, Ps. Marítim de la Barceloneta, 37-49, 08003 Barcelona, Catalunya, Spain; 2 National Institute of Aquatic Resources (DTU Aqua), Section for Marine Ecology and Oceanography, Technical University of Denmark, Kavalergården 6, DK-2920 Charlottenlund, Denmark; 3 King Abdullah University of Science and Technology (KAUST), Red Sea Research Center, Thuwal, Saudi Arabia; 4 Department of Biosciences, University of Oslo, Postboks 1066 Blindern, 0316 Oslo, Norway; 5 Department of Bioscience, Aarhus University, Frederiksborgvej 399, PO Box 358 DK-4000 Roskilde, Denmark; 6 Marin Microbiology, University of Bergen, Thormøhlensgate 53 B, 5006 Bergen, Norway; 7 Department of Biosciences & Centre for Sustainable Aquatic Research (CSAR), Swansea University, SA2 8PP, United Kingdom; 8 Uni Research Environment, P. box 7810, 5020 Bergen, Norway; 9 Remote Sensing Group. Plymouth Marine Laboratory, PL1 3DH, UK

## Abstract

Intensive sampling at the coastal waters of the central Red Sea during a period of thermal stratification, prior to the main seasonal bloom during winter, showed that vertical patches of prokaryotes and microplankton developed and persisted for several days within the apparently density uniform upper layer. These vertical structures were most likely the result of *in situ* growth and mortality (e.g., grazing) rather than physical or behavioural aggregation. Simulating a mixing event by adding nutrient-rich deep water abruptly triggered dense phytoplankton blooms in the nutrient-poor environment of the upper layer. These findings suggest that vertical structures within the mixed layer provide critical seeding stocks that can rapidly exploit nutrient influx during mixing, leading to winter bloom formation.

The oceanic surface mixed layer (ML) is defined as a layer where turbulent mixing has homogenised the water, and is typically characterised by quasi-uniform density profiles and, presumably, even distribution of phytoplankton. However, the fact that a layer has been actively mixed in the recent past does not imply mixing is occurring at all times, and even if turbulent mixing is ongoing this does not necessarily mean phytoplankton are homogeneously distributed^1^. In this sense, we should distinguish ML from the “mixing layer” where turbulent processes are actively acting and mixing the water. The ecological implications of vertical structures of phytoplankton within the ML are high. From a theoretical point of view, most of the bloom formation theories assume an even distribution and constant grazing mortality of plankton within the ML; therefore, ML depth should be a good proxy for modelling seasonal bloom formation^2^,^3^,^4^. Accordingly, the bloom initiates when the thermocline is shoaling in spring. This concept has been recently challenged and alternative theories exist now to incorporate patchiness into the bloom initiation in temperate and high latitude seas^1^,^5^. However, empirical evidence of heterogeneous phytoplankton distribution and activity within the ML is scarce^6^,^7^, and studies investigating the role of such patches on bloom initiation are absent.

We hypothesise that phytoplankton patchiness not only occurs and persists within the ML, but it is actually important for the onset of seasonal bloom, especially in warm oligotrophic seas where relatively calm weather conditions are common even in the blooming season, allowing for biological heterogeneity within the ML. In these areas, light is seldom limiting for phytoplankton growth, but nutrients are found at very low concentrations in surface waters most of the year. When nutrients are virtually absent in the ML, shoaling of ML would not trigger bloom formation. On the contrary, deepening of the ML may facilitate the access to nutrients from below and initiate the bloom.

From an ecological point of view, phytoplankton patches within the ML provide a haven in a resource-poor environment by favouring nutrient recycling, similar to oases in deserts. The aim of the present study was therefore to explore the presence and driving forces behind patch formation of planktonic organisms in the upper ML of the Red Sea (Fig. 1) and the implications for bloom initiation. We chose the central Red Sea as a model site for tropical oceans with marked density stratification and a deep ML^8^,^9^,^10^. A winter phytoplankton bloom^11^,^12^,^13^, coinciding with a decrease in temperature and deepening of the ML^11^,^12^,^14^, characterizes this area. We conducted our research in late autumn from 28 November to 5 December, which was prior to the winter bloom, to capture the pre-bloom community dynamics and to better understand the mechanisms behind this seasonal event. All major planktonic components of the food web were sampled during a period of calm weather. We argue that vertical patchiness of small plankton within the ML (here defined as the depth with a change of 0.125 in sigma-*t* from the surface^15^) was the result of a trade-off between nutrient use and grazing mortality.

## Results

### Central Red Sea seasonality and weather conditions

The central Red Sea weather conditions in 2012 were typical for the area; i.e., low barometric pressure and high temperature during July and the opposite during January (Fig. 2a,c; Upper Ocean Processes Group, Woods Hole Institution, USA, http://uop.whoi.edu/projects/KAUST). Wind speed averaged between 4 and 5 m s^−1^ (Fig. 2e). In weeks prior to our study there was an evident decline in average temperature, and a modest increase in wind speed few days before the first sampling (Fig. 2d,f). During our study, there was a subtle increase in temperature and decrease in wind speed (Fig. 2d,f). There was no precipitation throughout the sampling.

Maximum sea surface temperature (SST) occurred during summer and extended to early autumn (Fig. 3a). The temperature abruptly dropped in November, coinciding with the onset of seasonal phytoplankton bloom, which reached its maximum in February 2013 (Fig. 3a). Our sampling therefore coincided with the initial phase of the bloom (Fig. 3b) and a stable SST period between two periods of temperature drop.

### Vertical profiles

The temperature and salinity profiles showed distinct stratification at 50–80 m that demarcated the surface ML (Fig. 4). The euphotic zone (defined as the layer with ≥1% surface irradiance) extended only to 50 m (Fig. 5). Inorganic nutrients were high below the pycnocline, but were very low within the ML (Fig. 6).

There was a clear but modest chlorophyll *a* (hereafter chl *a*) maximum (<0.6 μg chl *a* L^−1^) between 10 and 60 m (Fig. 7), and chl *a* concentr*a*tion was considerably lower below the ML. During the study, the distribution of diatoms (mostly *Nitzschia* (*Ceratoneis*) *closterium*, *Chaetoceros* spp. and pennate diatoms) showed a deepening from 20 m to 80 m, where they occurred at ca. 10 cells mL^−1^ (Fig. 8). Dinoflagellates showed a similar, but less prominent, pattern, deepening from 10 m to 50 m after a week (Fig. 8). Phototrophic flagellates peaked mostly below the ML, at 80 m, showing also a maximum abundance on Day 4 at surface (Fig. 8). Ciliates occurred in highest concentrations in the upper 30 m at the beginning of the sampling period; the distribution was deeper at the end of the period, with maxima at 50 m by Day 8 (Fig. 9). Heterotrophic flagellate concentrations were variable in time, and showed a rather homogeneous vertical distribution pattern that extended to the thermocline and below, with the exception of a surface peak on Day 6 and a minimum at 70 m on Day 8 (Fig. 9). Autotrophic prokaryotes were mostly restricted to the ML and declined in abundance with depth, with peaks at 10–20 m on Days 2 and 7 (for both *Prochlorococcus* and *Synechococcus*; Fig. 10). Heterotrophic bacteria abundance was highest in the upper 60 m (Fig. 10). The abundance of this group was remarkably low below the thermocline, but increased again in deeper waters.

The coefficient of variation of the density distribution profiles within the ML according to our definition ranged 1–2%, whereas for the plankton groups considered ranged from 10 to 100% (mean 46%), with diatoms and *Synechococcus* being the most variable and heterotrophic flagellates and bacteria the less variable.

### Mesozooplankton vertical distribution

The distribution of mesozooplankton corresponds to a snapshot rather than the daily patterns of their vertical position (Table 1). However, because we sampled most of the ML, the integrated values can be used to calculate trophic impacts, as described in the discussion. The community was dominated by copepod nauplii and copepodites (mean abundance of 3 and 4 ind L^−1^, respectively), followed by appendicularians (mean 1.5 ind. L^−1^), and chaetognaths, which were 1–2 orders of magnitude less abundant.

### Phytoplankton growth and mortality rates

We aimed at conducting experiments with waters from the upper and lower sections of the ML. However, in the last two dilution grazing assays (Days 6 and 7) the deeper-water experiments were conducted with water from below the ML (as we defined). Nevertheless, according to the temperature and salinity profiles, these deep-waters did not differ much in their physical characteristics than the ones above. The experimental data on dilution grazing assays allow us to obtain both microzooplankton grazing estimates and phytoplankton growth rates, and to evaluate the extent of phytoplankton nutrient limitation. Phytoplankton instantaneous growth rates (μ_o_, chl *a*-based) were higher in the upper than in the lower ML, and at the beginning than at the end of the study period (Table 2). Conversely, microzooplankton grazing activity was less variable, with maxima in the lower ML (Table 2). The net growth rates of the different planktonic groups were also variable. Diatoms were growing in the upper ML during the entire study, but were declining (negative growth rate) in deeper layers (60–70 m) after Day 5 (Table 3). Dinoflagellates showed substantial growth only in the bottom of the ML on Day 4 (Table 3). For the rest of the flagellates (including both autotrophic and heterotrophic forms from Lugol counts), net growth rates were only positive on Days 5 and 6, and only at the surface (Table 3). Ciliates had high growth rates during the entire study in both strata except for two occasions (Day 5 at 20 m and Day 6 at 70 m) (Table 3).

Phytoplankton nutrient limitation was evaluated by comparing the net growth rates in nutrient amended bottles with the control (Fig. 11). Nutrient limitation was noted throughout the study and was more severe at higher phytoplankton growth rates in the upper ML samples.

### Simulation of mixing event

Mixing nutrient-rich deep water with ML-water triggered a phytoplankton increase from ca. 0.2 μg chl *a* L^−1^ to 4.5 μg chl *a* L^−1^ after six days, with most of the increase in the >10 μm fraction (Fig. 12a,b). Concurrently, phytoplankton in the control decreased by a factor of three. Flagellates were the dominant taxa in the treatment in the beginning, but diatoms eventually became the dominant constituent (Fig. 12c,d). In the control, there was a slight increase in ciliates and a decrease in dinoflagellates.

## Discussion

Using the temporal succession of depth profiles we have illustrated the presence of patchiness in the vertical distribution of planktonic organisms within the ML. We have also shown how different mechanisms may act to shape the structure of planktonic communities at different layers within the ML, and how the appropriate conditions may trigger a bloom in these nutrient-poor waters within a few days.

### The central Red Sea seasonal bloom

In many tropical marine ecosystems, like the Red Sea, phytoplankton growth in the euphotic layer is controlled primarily by the supply of nutrients (as light availability is high throughout the year). The Red Sea is considered a relatively oligotrophic basin (except the southern part)^11^,^16^. The relatively deficient reservoirs of nutrients in the Red Sea are trapped below the stratified zone, primarily due to the persistent pycnocline^14^. Lack of riverine input and negligible precipitation mean that phytoplankton in the nutrient-depleted ecosystem of the Red Sea rely principally on nutrients from the following sources: a) monsoonal-driven horizontal intrusion of nutrient-rich waters from the Indian Ocean^17^,^18^,^19^, b) nutrient recycling and mixing of water from below the nutricline^20^, c) the biologically-rich coral reefs^13^, and d) aerial depositions (e.g. dust storms) (hypothesised by Acker *et al.*^16^, but not yet confirmed).

For the central Red Sea, previous evidence suggests that deepening of the ML leads to winter phytoplankton bloom^11^,^12^,^14^. Although we do not have direct evidence of variations of the ML depth for 2012, it is accepted that changes in SST provide an indirect indication of vertical mixing, which may influence nutrient availability^21^ and hence phytoplankton bloom development. Remote sensing of chlorophyll has limitations especially in optically complex waters, where particulate and/or dissolved organic matter do not co-vary in a predictable manner with chlorophyll^22^. Therefore, chlorophyll data may be influenced (generally resulting in an overestimation) by the factors mentioned above, especially in the coastal waters and shallow reef-inhabited waters such as the Red Sea. However, not all the coastal high chlorophyll values are necessarily erroneous, as the large coral reef complexes may be sources of either nutrients or chlorophyll-rich detritus that enhance phytoplankton production near the reefs^16^. In fact, recent evidence clearly shows a significant relationship between satellite and *in situ* chloroplyll data in coral reef areas of the Red Sea^13^, including our study area. The scope of this study is to investigate the regional seasonal succession and timing of phytoplankton blooms, regardless of the absolute chlorophyll concentrations.

### Major drivers of plankton vertical structures in the Red Sea

The formation of biological thin layers due to physical processes or behavioural aggregations has been described in the literature 23,24,25,26. However, in our case, vertical segregation of non-migrating groups (prokaryotes, and protists) was evident in a physically uniform water body. What, then, are the mechanisms driving the formation of these vertical patches?

For motile mesozooplankton, ciliates and dinoflagellates, it is expected that their distribution will be influenced by behaviours such as foraging, avoiding predators, and so on. For organisms that actively control their buoyancy, such as diatoms, changes in their physiological rates due to, for instance, severe nutrient limitation would affect their vertical position in the water column^27^. This phenomenon was evident in the gradual deepening of diatoms observed in the course of our study. As shown in Table 3, the diatoms in the deeper strata were apparently dying. Nutritional resources (either inorganic or organic nutrients for autotrophs and prokaryotes, or prey for heterotrophs) are, therefore, one of the relevant factors to consider. Our data indicate that nutrient limitation occurred first in the upper ML then progressed downwards. Nutrient concentration in the deeper part of the ML, albeit low, was still higher than in the upper ML. However, in the lower strata, light was limiting (<1% surface PAR intensity) and compromising photosynthesis, which explains the negative phytoplankton growth rates (μ_o_ based on chl *a*) there. Some researchers have reported actively growing autotrophs at even deeper depths and lower light conditions in other oligotrophic oceans. However, in those cases, the chl *a* maximum w*a*s located below the ML, and nutrient inputs from the deeper water most likely allowed the phytoplankton to endure light limitation^28^,^29^,^30^. Given the deep thermocline in our study area, deep phytoplankton communities would likely not thrive under the combined stress of low light, low nutrients and high temperature.

Another important factor shaping the distribution of marine organisms is predation. We measured microzooplankton grazing on phytoplankton, and found different coupling between phytoplankton growth and grazing rates in different strata, with positive phytoplankton net growth rates in the upper ML. Vertical differences between phytoplankton growth rate and grazing mortality rate are also found in other studies, even within the ML^6^,^7^. To our knowledge, this has never been considered as a driver of phytoplankton distribution within the ML. The highest phytoplankton concentrations in the chl *a* maximum layer differed little from that in the surrounding waters, and could be easily attained in 24 h based on the observed phytoplankton growth and mortality rates.

The mesozooplankton community was dominated by copepods and appendicularians (Table 1). Copepods are unable to efficiently feed on, and therefore do not impact, picophytoplankton^31^,^32^,^33^. However, appendicularians can retain picoplankton and, with maximum filtration rates that may clear the water column on a daily basis^34^,^35^, have the potential to shape the vertical distributions of prokaryotes and protists. Appendicularians may position themselves in the water column to maximise encounter rates with their prey and thus benefit from patchy environments^36^,^37^. This behaviour would imply that their grazing activities would over time reduce patchiness of their prey (i.e., the “killing the winner” hypothesis^38^). Therefore, the actual potential for patch-formation by the winter microbial community in the central Red Sea should be much stronger than the one observed in this study.

### Potential for bloom formation

During the six-day incubation, chl *a* concentration increased 9 times to 4.5 μg chl *a* L^−1^ in bottles amended with deep water and the corresponding net growth rate was ca. 0.5 d^−1^. Hence, under a hypothetical mixing event with nutrient-rich waters from below the thermocline, our results suggest that a phytoplankton bloom can develop rapidly. The observed phytoplankton growth rates were both in the range of the net growth rates found for phytoplankton at 20 m in our first dilution grazing experiments (coinciding with the microcosm experiment) and in accordance with recent meta-analyses of microzooplankton grazing rates in the oceans^39^, but were much higher than the rates obtained by satellite data of the actual bloom in the area. This discrepancy may be due to several reasons: Firstly, we mixed equal amounts of deep nutrient-rich water with ML water in a short period of time, which does not mimic the natural mixing process that lasts some days and is more gradual. Secondly, we removed larger than 50 μm grazers, who could be crucial for regulating phytoplankton growth and bloom development.

Several conventional bloom formation theories have been discussed in the literature. According to Sverdrup’s critical depth hypothesis (CDH)^2^, spring-time increase in irradiance and shoaling of the ML provide favourable conditions for phytoplankton bloom development. The CDH assumes even distribution of organisms within the ML and non-limiting nutrients, which evidently is not true for the central Red Sea. The critical turbulence hypothesis (CTH)^40^ offers an alternative explanation of bloom formation even in the absence of vertical stratification, but it assumes a constant grazing mortality of phytoplankton within the ML, which also is inconsistent with observations^6^^, (this study)^. More recently, the disturbance-recovery hypothesis (DRH)^41^ asserts that blooms are initiated when the dilution effect from ML deepening ceases. The central Red Sea does not seem to follow this pattern because the bloom usually occurs when the thermocline is deepening^11^,^12^,^14^^, (this study)^, with nutrients mainly entering the ML gradually from below and light being seldomly limiting during the year.

Our results offer then a different view of seasonal bloom formation in tropical oligotrophic seas such as the Red Sea, where phytoplankton standing stock is low and the weather conditions are relatively calm in winter. We propose that patchiness of organisms may persist in the winter ML and the dilution effect of the deepening of the ML is irrelevant for these environments. To the contrary, deepening ML entrains any deep chl *a* from below it. Under these conditions, bloom may occur at any time, as long as there is enough water column stability, nutrients and light to allow the accumulation of organisms. Once a critical seed population is reached, any further mixing will fertilise the ML with nutrients to generate a bloom. As has been hypothesised, blooms are loopholes in zooplankton grazing^42^, and, even though grazing may remain high, phytoplankton only need to disrupt the predator–prey equilibrium to bloom^38^,^39^,^41^. Our observations provide an alternative explanation for phytoplankton bloom formation in some warm oligotrophic seas where conventional bloom formation theories fall short, and will help guide future field observations and sampling strategies to better understand bloom dynamics in those regions.

## Materials and methods

### Remotely sensed datasets

The weekly (8-days composite) satellite remotely-sensed ocean colour (chlorophyll) and night-time SST dataset were acquired from NASA Ocean Biology Processing Group (OPBG, http://oceancolor.gsfc.nasa.gov/) on the coordinates Latitude 22.36°N to 22.2°N, and Longitude: 38.8°E to 39°E. The Moderate-resolution Imaging Spectroradiometer (MODIS on-board the Aqua platform) 4 km resolution data were processed for the period 2012-2013. Standard NASA algorithms were used for the near-surface Chl *a* (OC3) estimates, which are routinely processed by the OPBG at the Goddard Space Flight Center^43^. A recent comparison between MODIS chlorophyll and *in situ* datasets clearly indicated that the performance of the standard NASA chlorophyll algorithm is comparable to other oligotrophic regions in the global ocean, supporting the use of satellite ocean colour in the Red Sea^44^.

### Sample collection and analyses

**Figure 1:**
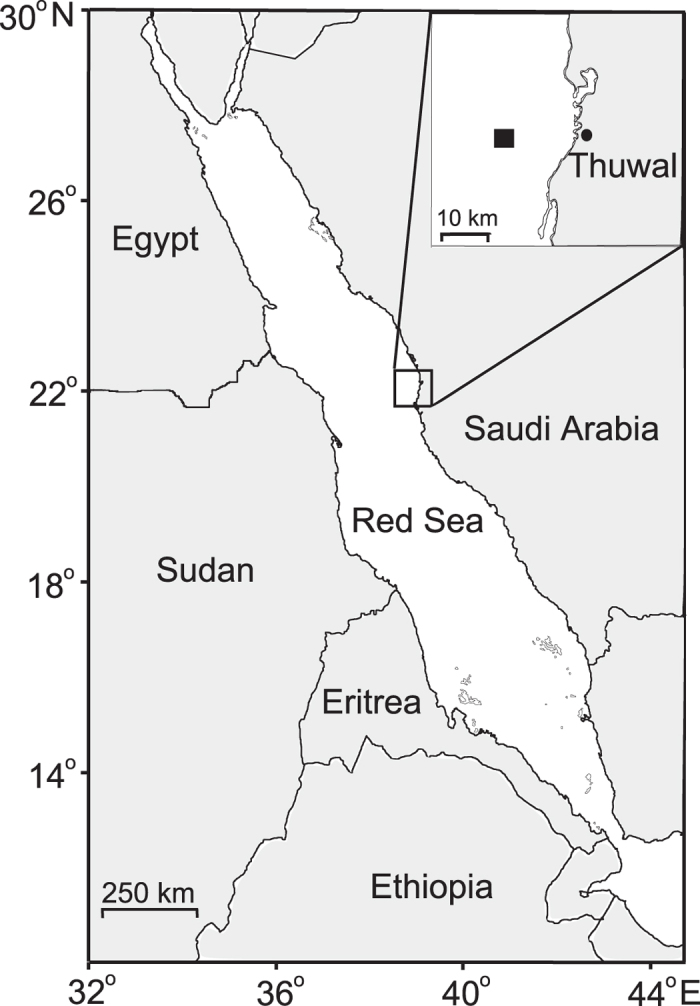
The study area showing the position of the sampling station. The map was generated with Inskape 0.48.5 software from Software Freedom Conservancy.

**Figure 2:**
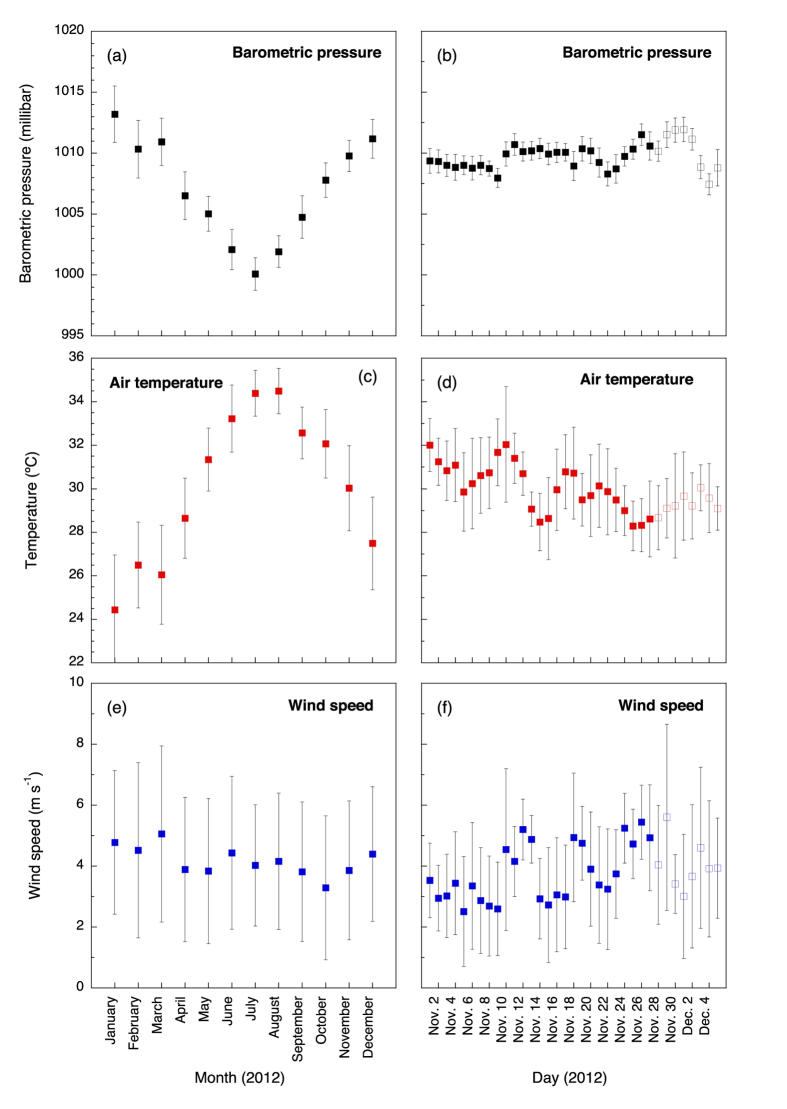
Time series of the climatological conditions for the area of study (barometric pressure, air temperature, and wind speed). Left panels (**a**,**c**,**e**) show the data for the year of the study (2012). Right panels show in further detail the data for the weeks preceding the sampling (dark symbols) and for the sampling period (open symbols). Error bars are SD.

**Figure 3:**
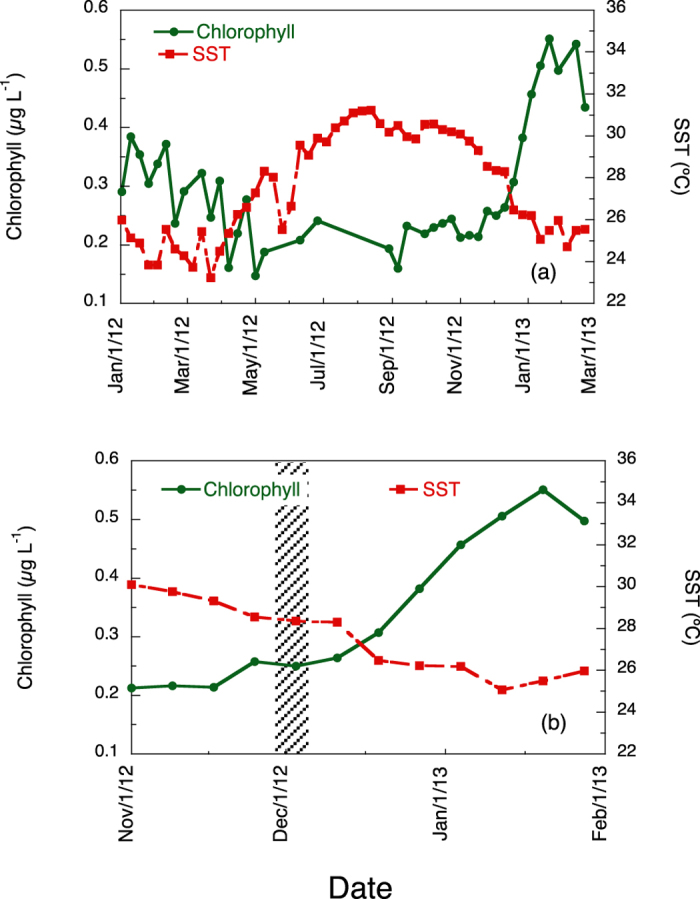
Time series of the remote sensed sea surface temperature (SST) and chlorophyll for the area of study. (**a**) data from January 2012 to March 2013. (**b**) detail of the conditions around the dates of the study (indicated by the shaded vertical bar).

**Figure 4:**
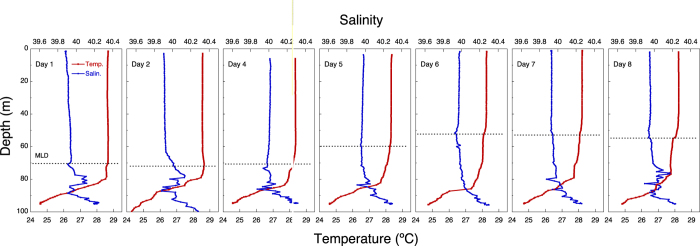
Time series of the vertical profiles of temperature (°C) and salinity. The discontinuous horizontal line indicates the depth of the ML.

**Figure 5:**
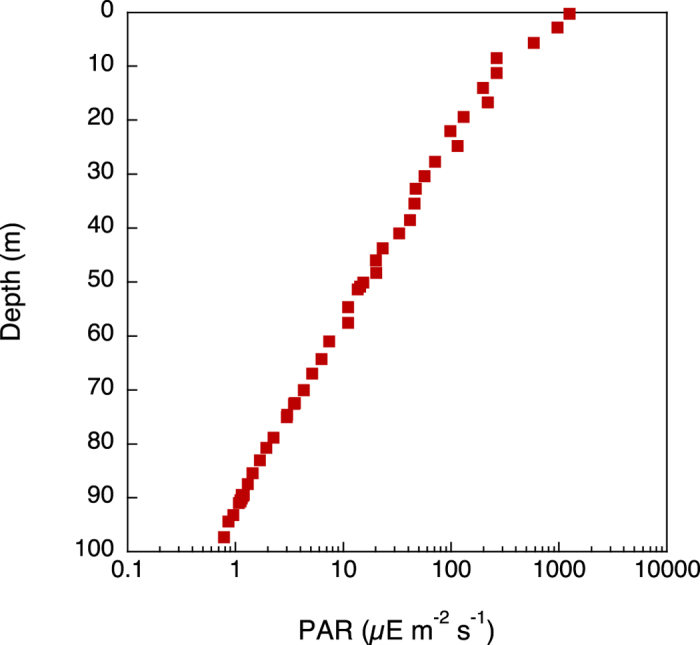


**Figure 6:**
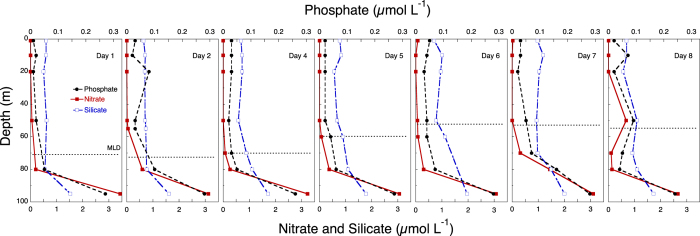
Time series of the vertical profiles of nitrate, phosphate, and silicate in μmol L^−1^. The discontinuous horizontal line indicates the depth of the ML.

**Figure 7:**
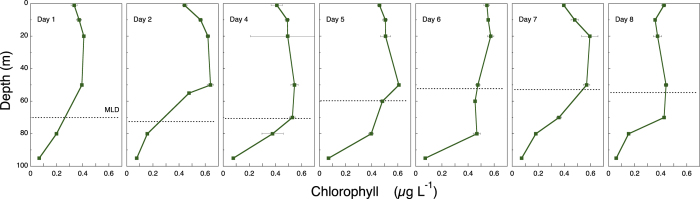
Time series of the vertical distribution of chlorophyll *a* (chl *a*) in μg L^−1^. The discontinuous horizontal line indicates the depth of the ML.

**Figure 8:**
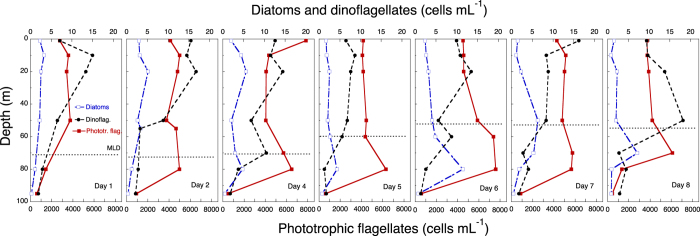
Time series of the vertical distribution of autotrophic protists: diatoms, dinoflagellates, and phototrophic flagellates in cells mL^−1^. The discontinuous horizontal line indicates the depth of the ML.

**Figure 9:**
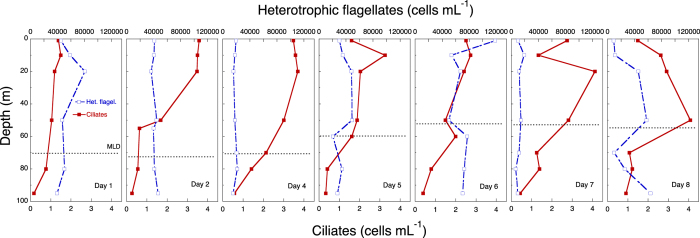
Time series of the vertical distribution of protozoans. Hetrotrophic flagellates and ciliates in cells mL^−1^. The discontinuous horizontal line indicates the depth of the ML.

**Figure 10:**
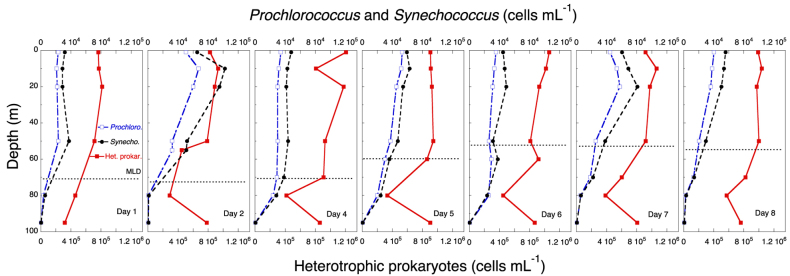
Time series of the vertical distribution of prokaryotes in cells mL^−1^, *Prochlorococcus* spp., *Synechococcus* spp., and heterotrophic prokaryotes. The discontinuous horizontal line indicates the depth of the ML.

**Figure 11:**
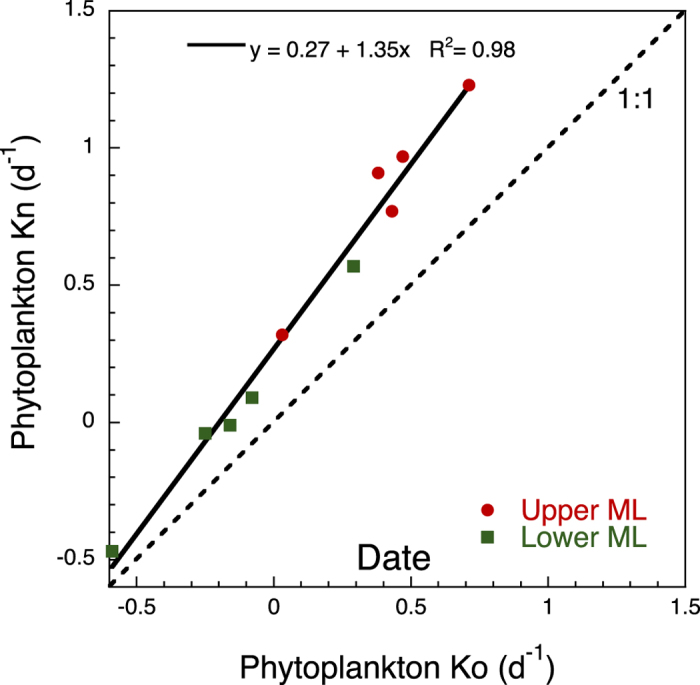
Phytoplankton nutrient limitation. Regression line of the phytoplankton net growth rates (chl *a*-based) without added nutrients (K_o_; d^−1^) against those with added nutrients (K_n_; d^−1^) from dilution grazing experiments. Dots correspond to samples from the upper ML; squares correspond to samples from the lower ML. Dotted line indicates the 1:1 relationship.

**Figure 12:**
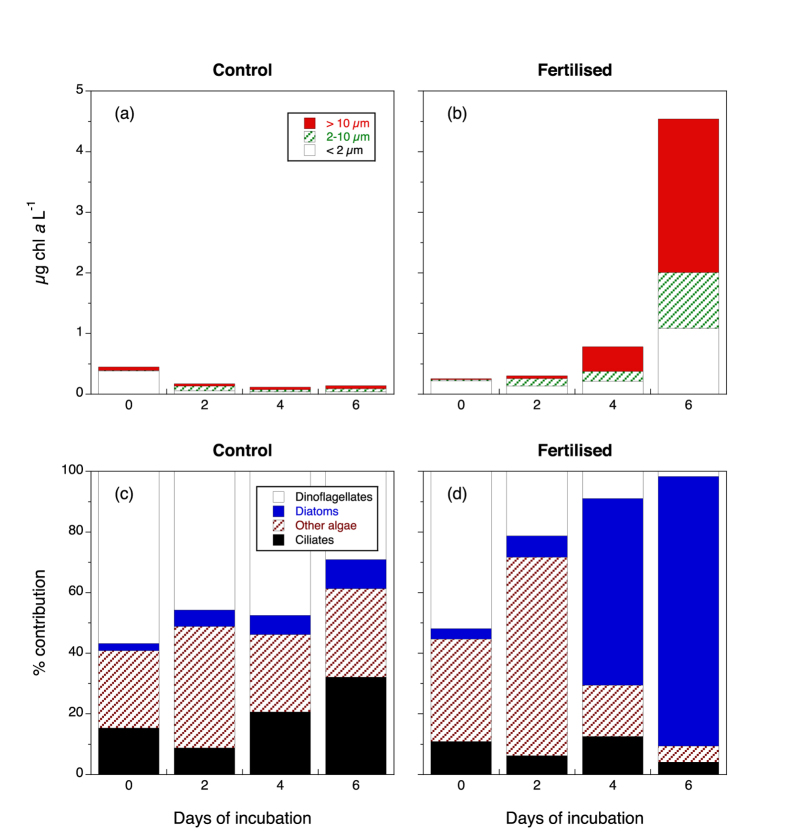
Upper panels: Chl *a* concentration (μg L^−1^) during the microcosm incubations for the artificial bloom generation experiment. Three chl *a* size fractions are presented. (**a**) Control bottles containing water from 10 m. (**b**) Fertilised bottles containing water from 10 m mixed with an equivalent amount of water from below the thermocline. Lower panels: Proportion (volume-based) of the dominant protists from microscopic Lugol counts during the microcosm incubations for the artificial bloom generation experiment. (**c**) Control bottles containing water from 10 m. (**d**) Fertilised bottles containing water from 10 m mixed with an equivalent amount of water from below the thermocline.

From November 28th to December 5th 2012 (Day 1 to Day 8), we sampled a coastal station located at 22.28072° N 38.93709° E, approximately 15.5 km from the coast of Thuwal, Saudi Arabia, with an average depth of 120 m (Fig. 1). Every day (except for November 30th, Day 3) at noon we obtained profiles of salinity and temperature with a CTD (Idronaut 316plus). ML depth was calculated as the depth at which a change from the surface sigma-*t* of 0.125 has occurred^15^. Light profiles of spectral irradiance (mW m^−2^ nm^−1^) were obtained with a Ramses hyperspectral radiometer (SAMIP_ACC_VIS - Hyperspectral UV-VIS Irradiance sensor- 320–950 nm) on Day 5 and converted to μmol m^−2^ s^−1^ nm^−1^ using the MSDA_XE Software. To calculate PAR for each depth, all measurements were added and multiplied by the wavelength interval (3.34 nm). Water samples for inorganic nutrients, chl *a* concentrations, flow cytometry, and microscopic counts were obtained daily at 10–20 m intervals using a rosette with 5 L Niskin bottles. Dissolved inorganic nutrient samples (phosphate, nitrate, and silicate) were immediately frozen (−20 °C) for later analysis on a Skalar autoanalyser (Breda, Netherlands) following the procedures of Hansen and Koroleff^45^. The precision (analytical reproducibility) of the nutrient analyses was 0.06, 0.1, and 0.2 μM for phosphate, nitrate, and silicate, respectively. Chl *a* concentration was determined by filtering 250 mL of water through GF/F Whatman filters. The filters were immersed in 5 mL 96% ethanol in the dark at room temperature for 12–24 h^46^. Fluorescence was then measured before and after acidification on a Turner TD-700 fluorometer (Turner Designs, California, USA) calibrated against chl *a* standard. Heterotorphic prokaryotes, small phytoplankton including prokaryotic *Prochlorococcus*, *Synechococcus*, and eukaryotic phototrophic flagellates, and heterotrophic flagellates were enumerated using a FACS Calibur (Becton Dickinson, Oxford, UK) flow cytometer. Triplicate 2 mL samples were fixed with glutaraldehyde (0.5% final concentration) for 2 h in the dark at 4 °C and thereafter flash-frozen in liquid nitrogen and stored until analysis (<4 months) at −80 °C. Phytoplankton groups were discriminated on the basis of their side-scatter and their pigment fluorescence. Heterotrophs were stained with SYBR Green I (Molecular Probes Inc., Eugene, Oregon) and discriminated on biparametric flow cytometry plots for virus and prokaryotes^47^ and heterotrophic flagellates^48^.

The abundance and taxonomic composition of microplankton were obtained by microscopic examination of acid Lugol-preserved samples (2% final concentration). The samples were settled in 100 mL Utermöhl chambers for at least 48 h prior to counting using an inverted microscope (XSB-1A). The whole chamber, or a fraction of it for the smallest and more abundant organisms, was counted at 100, 250, and 400X magnification, depending on the group. Fifty to one hundred cells per group were sized and adjusted to their closest geometric shape, to obtain volume estimates.

Note that the flagellate samples were processed in two ways: for vertical profiles, flow cytometer was used to distinguish the flagellates by size (pico- and nano-sized) and by trophic role (photo- and hetero-trophic), whereas for the microcosm experiments, the flagellate samples were preserved in Lugol’s solution, microscopically sized and converted to biomass^49^.

Mesozooplankton were collected daily, ca. every 20 m, in two casts with the rosette holding three 10 L Niskin bottles (total of 60 L per stratum sampled). The water from the bottles was gently siphoned into 20 L containers. Immediately afterward, the mesozooplankton were concentrated on a 15-μm sieve, rinsed into a bottle, and fixed in 2% Lugol’s solution. The samples were counted and identified using an Olympus dissection microscope.

### Dilution grazing experiments

We conducted dilution grazing experiments^50^ with water from depths of 20 m (defined as upper ML) and 50–70 m (lower ML) five times over the sampling period, from November 28 to December 5 2012 (Days 1, 4, 5, 6 and 7). Natural seawater (NSW) for the experiments was collected using 5 L Niskin bottles in a rosette. A portion of the water was gently siphoned through a silicone tube into an acid-washed dark polycarbonate container, while the rest was filtered through a 0.2 μm PolyCap capsule using a peristaltic pump. The filtered seawater (FSW) was added to a series of 2.3 L polycarbonate bottles in pre-determined volumes. The remaining volume of the bottles was gradually (1/4 at a time) filled with unfiltered NSW by reverse filtration through a 224-μm filter to obtain a dilution series of 25, 50, 75 and 100% NSW in duplicate. Nitrate, phosphate, and silicate (2.5 mL of B1 media and 2.5 mL silicate working solution^51^ per incubation bottle) were added to the bottles, except for two bottles containing undiluted NSW (controls). Start samples were taken both for chl *a* (see above) and microplankton (250 mL) determination (see above). We also filtered 2 L of the experimental water through a 30-μm sieve to determine that large mesozooplankton were absent and only a few (ca. 5 ind. L^−1^) small copepodites (CI-III; <150 μm prosome length) were present in the experimental bottles.

Experimental bottles were incubated *in situ* at the corresponding experimental depths for approximately 24 h. Following incubation, samples were taken for chl *a* and microplankton from all bottles. Furthermore, to estimate the naturally occurring net growth rates of microplankton, we also took end samples for microscopic counts from bottles containing 100% NSW without nutrients. The samples were preserved in 2% Lugol’s solution for later analysis. For all experiments, the net phytoplankton growth rate (K), estimated from changes in chl *a* concentration during the incubation period, was plotted against the fraction of undiluted water, and model I linear regression was fitted to the data to obtain the slope (*m*; grazing mortality rate, d^**−**1^) 50. In two cases (Day 5 lower ML, and Day 7 upper ML) we found saturated feeding responses^52^, and linear regression was fitted only to the highly diluted treatments to obtain the phytoplankton instantaneous growth rates with added nutrients (*μ*), deriving *m* from there^53^.

### Simulation of mixing event

To assess the phytoplankton blooming potential in response to a mixing event, a simulation experiment was conducted by mixing equal parts of <50 μm filtered water from a depth of 10 m with water from a depth of 100 m (below the nutricline). Triplicated PC bottles (25 L), containing water from 20 m (control) or the mixed water (treatment), coinciding with a chl *a* patch, were incubated *in situ*, fixed at a depth of 20 m under a buoy system anchored at the study site. We sampled from the bottles every two days for six days. Initial and final samples of both microplankton and total chl *a* were obt*a*ined as described above. The fractions >2 μm and >10 μm chl *a* were obtained by filtering 500 mL onto polycarbonate Nuclepore^TM^ filters with pore sizes of 2- and 10-μm, respectively, and processed as above.

## Additional Information

**How to cite this article**: Calbet, A. *et al.* Heterogeneous distribution of plankton within the mixed layer and its implications for bloom formation in tropical seas. *Sci. Rep.* **5**, 11240; doi: 10.1038/srep11240 (2015).

## Figures and Tables

**Table 1:**
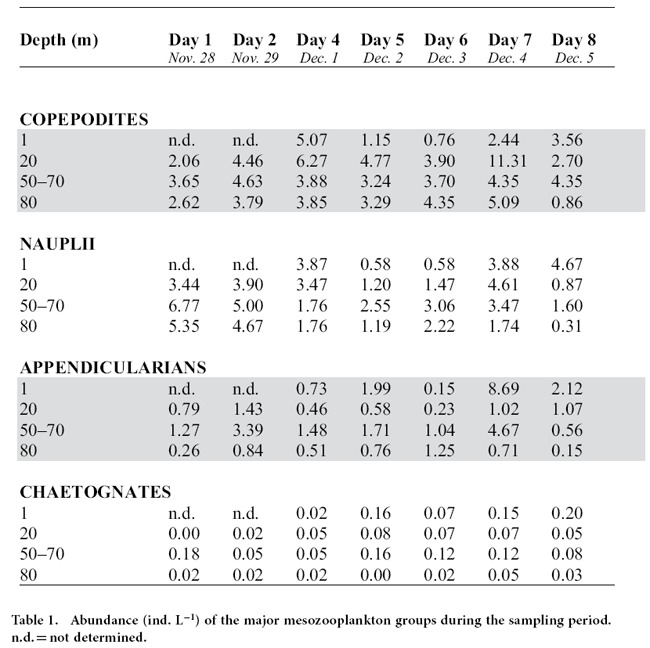
Abundance (ind. L^−1^) of the major mesozooplankton groups during the sampling period. n.d. = not determined.

**Table 2:**
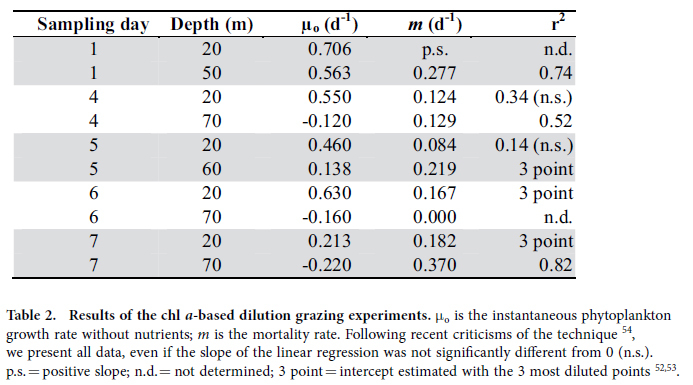
Results of the chl *a*-based dilution grazing experiments.

**Table 3:**
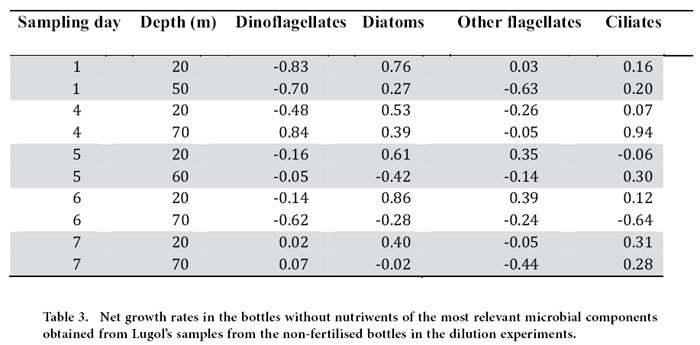
Net growth rates in the bottles without nutriwents of the most relevant microbial components obtained from Lugol’s samples from the non-fertilised bottles in the dilution experiments.
